# Effects of Caffeine Supplementation on Cognitive Function and Neuromuscular Performance Across Menstrual Cycle Phases in Eumenorrheic Female Athletes: A Randomized, Double-Blind Placebo-Controlled Pilot Trial

**DOI:** 10.3390/nu18101512

**Published:** 2026-05-09

**Authors:** Ines Ben Hsen, Sirine Hamdi, Halil İbrahim Ceylan, Siwar Erriahi, Andrea de Giorgio, Ismail Dergaa, Nicola Luigi Bragazzi, Mohamed Amine Bouzid

**Affiliations:** 1 Research Laboratory: Education, Motricité, Sport et Santé, EM2S, LR19JS01, High Institute of Sport and Physical Education, University of Sfax, Sfax 3000, Tunisia; 2 Department of Physical Education of Sports Teaching, Faculty of Sports Sciences, Atatürk University, Erzurum 25040, Türkiye; 3 Artificial Engineering, 80121 Naples, Italy; andrea@degiorgio.info; 4 Department of Biological Sciences, High Institute of Sport and Physical Education of Ksar Said, University of Manouba, Manouba 2010, Tunisia; 5 Department of Clinical Pharmacy, Saarland University, 66123 Saarbrücken, Germany

**Keywords:** menstrual cycle phases, physical performance, eumenorrheic female athletes, neuromuscular performance, cognitive function, countermovement jump, caffeine supplementation

## Abstract

**Background**: Hormonal fluctuations across the menstrual cycle may influence cognitive and neuromuscular performance in female athletes. Caffeine (CAF) is a widely used ergogenic aid, yet its phase-specific effects remain unclear. This study investigated the acute effects of CAF supplementation on cognitive and physical performance across menstrual cycle phases in eumenorrheic female athletes. **Methods**: Twelve trained female athletes with regular menstrual cycles participated in a randomized, double-blind, placebo-controlled study. Each participant completed a battery of cognitive (reaction time [RT], vigilance test [VT]) and physical performance tests (countermovement jump [CMJ], repeated sprint test [RST], and time to exhaustion test [TTE]) during the early follicular (EFP), late follicular (LFP), and mid-luteal (MLP) phases. CAF (400 mg) or a placebo (PLA) was ingested one hour before the testing session. **Results**: CAF supplementation significantly improved VT performance across all menstrual cycle phases compared with PLA (*p* < 0.05), with no phase-dependent effect. RT was significantly reduced following CAF ingestion (*p* < 0.05), with no significant condition × phase interaction. CMJ performance varied across menstrual phases under placebo conditions, with higher values observed during the LFP (*p* < 0.05); a trend toward enhanced jump performance was observed following CAF ingestion, particularly during the MLP. During RST, performance declined across sprints in all conditions (*p* < 0.05), and CAF supplementation attenuated fatigue-related performance declines during later sprints, irrespective of menstrual cycle phase. Time to exhaustion was not significantly influenced by CAF supplementation or menstrual cycle phase (*p* > 0.05). **Conclusions**: CAF supplementation was associated with consistent improvements in vigilance and reaction time across the menstrual cycle. However, effects on neuromuscular performance were less consistent and not clearly phase-dependent. These findings highlight that while CAF can enhance certain aspects of cognitive performance in female athletes, responses in physical performance may vary and require further investigation. CAF may contribute to improvements in selected neuromuscular outcomes, although evidence for phase-specific ergogenic effects remains limited.

## 1. Introduction

The menstrual cycle (MC) is a fundamental biological rhythm in females, second only to the circadian rhythm [1]. Governed by the hypothalamic–pituitary–ovarian axis, it involves a complex interplay of hormones including the gonadotropin-releasing hormone, follicle-stimulating hormone, luteinizing hormone, estrogen (E2), and progesterone (P4), which regulate reproductive physiology and influence neuromuscular, cardiovascular, thermoregulatory, and cognitive functions [2]. Typically lasting 28 days (range: 21–35 days), the MC begins with menstruation, followed by the follicular phase, ovulation, and the luteal phase, characterized by rising P4 levels, preparing the endometrium for potential implantation. If fertilization does not occur, P4 levels decline, the endometrium breaks down, and a new cycle begins [3,4,5,6].

Unlike male athletes whose endocrine environment is relatively stable, female athletes experience cyclical variations in hormone levels that can affect performance consistency. E2 is generally considered to exert neuroexcitatory effects, enhancing glutamatergic transmission and cortical excitability [7]. These hormonal dynamics may influence a range of performance parameters, including reaction time, endurance capacity, agility, cognitive flexibility, and mood [8,9,10]. E2 and P4 exert opposing effects on neural excitability, with E2 enhancing synaptic transmission and cortical excitability, while P4 tends to inhibit neural activity and motor coordination. These hormonal fluctuations across the MC may therefore influence both cognitive and neuromuscular performance in female athletes.

Such variations are particularly relevant among elite female athletes, who are increasingly participating in high-performance sports with rigorous physical demands. Environmental and lifestyle stressors, including intense training, low energy availability, psychological stress, and insufficient recovery, can further disrupt menstrual function, potentially leading to menstrual irregularities or energy deficiency syndromes [11]. Thus, tailoring training, recovery, and supplementation protocols to MC variation has emerged as a key strategy in sports science and female-specific performance optimization.

Caffeine (CAF), as a non-selective adenosine receptor antagonist, increases central nervous system excitability and reduces perceived fatigue, potentially counteracting the inhibitory effects of P4 and augmenting the excitatory influence of E2. Understanding these interactions is essential to elucidating how CAF supplementation might differentially affect performance depending on the MC phase (MCP).

In recent decades, numerous studies have highlighted the effects of CAF on physical performance. CAF is one of the most consumed psychoactive substances, often used for its beneficial effects on both physical and cognitive performance [12,13]. The existing body of scientific literature indicates that CAF supplementation improves cognitive functions, including reaction time, alertness, executive function, and mood, across various populations, including women of different ages [14,15,16,17]. Additionally, CAF is considered an effective ergogenic aid that enhances memory, visuospatial reasoning, and reaction time, particularly in older populations [15]. Despite the steady rise in female participation in sports over the last two decades, research on strategies to mitigate MC’s influence on performance remains limited. Interestingly, female athletes may exhibit greater sensitivity to CAF than men [18], which may be attributed to physiological (e.g., hormonal fluctuations) or psychosocial factors (e.g., differences in consumption habits).

Despite increasing recognition of sex- and gender-specific differences in exercise physiology and pharmacology, the interaction between CAF supplementation and MCP-specific performance remains underexplored. While some studies report minimal differences in exercise capacity across MCPs, others highlight phase-dependent alterations in strength, fatigue, and cognitive function, raising questions about optimal CAF timing and dosage relative to the MC [19,20]. Addressing this knowledge gap is essential for advancing evidence-based nutritional strategies that align with the physiological realities of female athletes.

Therefore, the current study aimed to investigate the acute effects of CAF supplementation on cognitive and physical performance across the MCPs in trained eumenorrheic female athletes. We hypothesized that CAF intake would enhance both cognitive and physical performance across all MCPs, with possible MCP-specific variations reflecting underlying hormonal influence.

## 2. Material and Method

### 2.1. Participants

This randomized, double-blind, placebo-controlled trial was designed and reported in accordance with the “Consolidated Standards of Reporting Trials” (CONSORT) guidelines (Supplementary Materials) [21]. Twenty-five female athletes (age: 24.4 ± 2.7 years, height (m) 1.62 ± 0.05) volunteered to participate in this study (Table 1).

The athletes carried out five training sessions per week and participated in weekly competitions (1 match per week). The present study was approved by the Regional Research Ethics Committee (CPP SUD N° 0442, registration date: 6 September 2022), registered in the Pan African Clinical Trial Registry (PACTR202602512638897), and conducted in accordance with the ethical principles of the Declaration of Helsinki.

**Figure 1:**
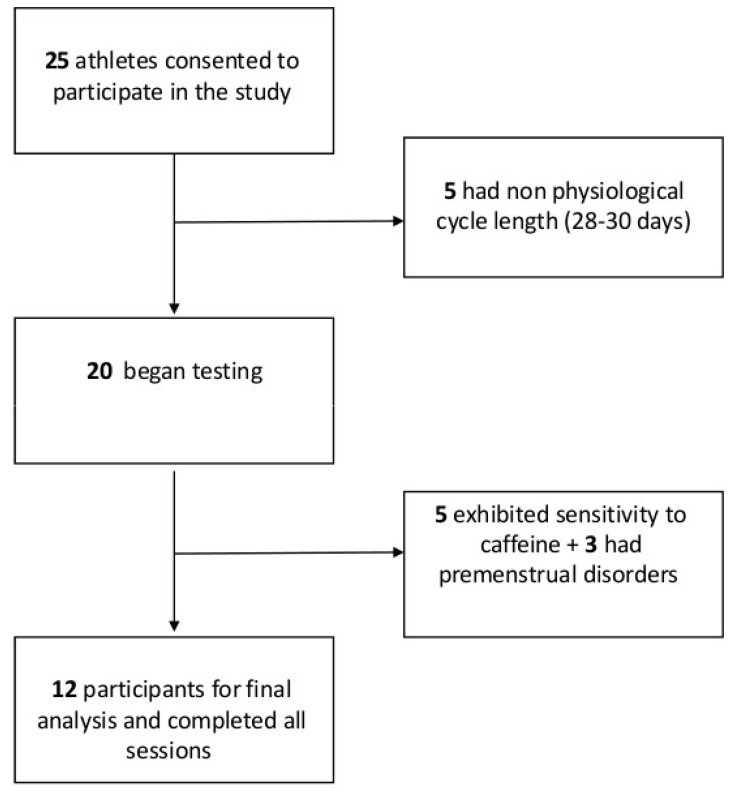
Participant flow diagram of the study.

The inclusion criteria for participation in the study were: (a) the absence of any form of contraception (oral, implanted, injected, intrauterine devices, or patches); (b) a regular MC of physiological length (28–30 days) [16]; (c) being free from any illness or disease that could affect performance and health; and (d) not suffering from an injury that would affect their performance. Therefore, 12 subjects were suitable for analysis (Figure 1).

A formal sample size calculation was not conducted due to the exploratory nature of this study and the limited availability of trained eumenorrheic female athletes meeting the inclusion criteria. Consequently, the study included all eligible participants who consented to take part, resulting in a final sample of 12 athletes. This approach is consistent with similar exploratory studies in female athlete populations [22].

After being briefed on all experimental procedures, associated risks, and potential benefits, the athletes gave their consent to participate. Each participant completed questionnaires to confirm prior supplement use and the amount of CAF consumed daily [19], as well as to assess their medical history and physical activity level (International Physical Activity Questionnaire [23]). Participants who disclosed the use of medicinal substances, nicotine, or treatments that might obscure CAF effects, or who exhibited sensitivity to CAF, were excluded from the study. Regular CAF consumption was defined as approximately 100 mg per day, equivalent to one cup of instant coffee [24].

### 2.2. Study Design

The experimental protocol involved three consecutive testing sessions, each conducted during a distinct phase of the MC, including the early follicular phase (EFP), late follicular phase (LFP), and mid-luteal phase (MLP), with a 7–10-day rest period between visits. Each participant completed two experimental sessions per MCP: one with CAF and one with placebo (PLA), resulting in a total of six sessions per participant. These sessions were conducted across multiple MCs to allow sufficient washout between treatments. A 7–10-day washout period was maintained between sessions to minimize potential carryover effects. The order of conditions (CAF or PLA) was randomized and double-blinded for each participant. During each MCP, participants completed a battery of assessments across two experimental conditions: a PLA session and a CAF session. Participants were instructed to abstain from all CAF-containing products for 24 h prior to each experimental session. Compliance was verbally confirmed at the start of each session. Additionally, participants were asked to report any symptoms potentially associated with CAF withdrawal, although no significant withdrawal effects were observed during the study. In the CAF condition, participants ingested 400 mg of CAF in capsule form, and in the PLA condition, an identical placebo capsule was administered. In both sessions, the supplement was taken 1 h before testing. CAF was administered at a fixed absolute dose of 400 mg. Based on participant body mass, this corresponded to an average relative dose of approximately 6.9–7.1 mg/kg across MCPs (Table 1). This dose was chosen based on previous literature showing ergogenic benefits in athletes at similar doses [25,26].

Cognitive performance was evaluated using a reaction time test (RTT) and a vigilance test (VT). In contrast, physical performance was assessed using a counter-movement jump (CMJ) test, a repeated sprint test (RST), and a time-to-exhaustion (TTE) test, all conducted at 90% of each subject’s maximal aerobic power.

### 2.3. Assessment of Menstrual Cycle Phases

Twice a month, participants completed a menstrual diary, documenting the date of menstruation, the duration and intensity of bleeding, and any associated discomfort. This data was collected for at least 6 months to accurately characterize the athletes’ MCs [15]. By retrospectively analyzing the data (counting back from the previous menstruation), the next phase of the MC was prospectively determined. Ovulation was confirmed by using ovulation test strips on midstream urine samples starting 3 days before the expected ovulation date. Additionally, serum levels of estrogen and progesterone were measured to confirm normal reproductive function and to ensure that test timing aligned with the MCP [27].

### 2.4. Assessments of Cognitive Function

#### 2.4.1. Vigilance Test

VT is a simple and validated measure of sustained attention. Participants were instructed to identify and cross out two specific target digits (i.e., 9 and 6) from a sheet containing a total of 600 random digits arranged across 36 horizontal lines. The task was to scan the digits line by line, from left to right, and mark the target digits as quickly and accurately as possible within 1 min. All non-target digits were to be ignored. The total number of correctly crossed-out target digits was recorded and used as the primary measure of vigilance performance for each participant [22].

#### 2.4.2. Reaction Time Test

RTT is a simple and reliable tool for assessing cognitive processing speed and response efficiency. In this study, visual reaction time was measured using the “Reaction” program (INRP 2005–2009, F. Jauzien, version 4.05). Participants were seated approximately 0.4–0.5 m from a computer screen. After completing 10 familiarization trials, the test began, using a blue square as the visual stimulus. Participants were instructed to press a designated keyboard key with their preferred hand as quickly as possible upon the appearance of the blue square. The final reaction time score was calculated as the average of 10 valid trials. Reaction times shorter than 150 milliseconds (indicative of anticipation) and longer than 800 milliseconds (suggesting lapses in attention) were excluded from the analysis to ensure data reliability [28].

### 2.5. Assessments of Physical Performance

#### 2.5.1. Repeated Sprint Test

Participants began the session with a standardized warm-up consisting of 5 min of cycling at a cadence of 60–80 revolutions per minute (rpm), followed by two 15 s progressive accelerations on a friction-loaded cycle ergometer (Monark 874E, Stockholm, Sweden).

The sprint protocol comprised 10 repeated sprints, each lasting 5 s, with 25 s passive rest intervals between efforts. A braking resistance of 0.9 N·kg^−1^ of body mass was applied to the flywheel to ensure a consistent load [29]. Participants were instructed to perform each sprint at maximal intensity from the start and were given verbal encouragement throughout to sustain maximal effort. They remained seated throughout both the sprint and recovery periods. Peak power was automatically calculated using the manufacturer’s validated software associated with the device. The software determines mechanical power by integrating force applied to the flywheel system, rotational resistance, displacement, and time variables during each movement. This integrated calculation provides an accurate estimate of instantaneous and peak mechanical power output expressed in watts (W).

#### 2.5.2. Counter-Movement Jump

Vertical jump performance was assessed using the OptoJump Next System (Microgate, Bolzano, Italy). For standardization, subjects began each trial in an upright position with their hands placed firmly on their hips, thereby eliminating the contribution of arm swing. Participants were then instructed to execute a rapid eccentric knee flexion (~90°) followed immediately by a maximal concentric vertical jump. This method evaluates explosive lower-limb power and is widely used in both athletic and research contexts [30].

#### 2.5.3. Time to Exhaustion Test

Participants first completed a 2 min warm-up on a cycle ergometer at an individualized workload of 1.5 W/kg body mass. Following the warm-up, they began the main test, cycling at 90% of their previously determined peak power output (mean ± SD: 304 ± 46 W).

Each participant was allowed to choose a preferred pedaling cadence between 60 and 90 rpm and was instructed to maintain it throughout the test. Visual feedback (on-screen display) and verbal encouragement were provided to help sustain consistent effort. The test continued until task failure, defined as a drop in cadence to below 70% of the initial self-selected value for at least 5 s. Throughout the trial, participants remained seated to minimize variations in muscle recruitment patterns. All tests were supervised by the same investigators, who provided standardized verbal motivation.

### 2.6. Perceived Wellness Assessment

Each morning at the same time, and during each MCP prior to the testing session, participants rated their perceived wellness using a modified version of the Hooper Index (HI) scale [31]. HI consists of four items: fatigue, stress, delayed onset muscle soreness (DOMS) (1 = very, very low; 7 = very, very high), and sleep quality (1 = very, very bad; 7 = very, very good), each rated on a 1–7 Likert scale. Higher scores for fatigue, stress, and DOMS indicate greater perceived strain, whereas higher scores for sleep quality indicate better recovery status.

### 2.7. Statistical Analyses

Statistical analyses were conducted using Statistica version 10 software (Statsoft, Tulsa, OK, USA). Data are presented in the text and tables as mean ± standard deviation (SD) and in the figures as mean ± standard error (SE). Given the small sample size employed, the normality of the distribution was assessed using the Shapiro–Wilk test, and all variables were found to be normally distributed. Following confirmation of normality, parametric tests were applied. For TTE, CMJ, VT, and RTT values were analyzed using a two-way analysis of variance (ANOVA) [Condition (PLA vs. CAF) × MCP (EFP vs. LFP vs. MLP)]. The peak power observed during each sprint was examined using a three-way repeated-measures ANOVA (Sprint time × MCP × Condition). For each statistically significant main factor effect and interaction effect, a post hoc Bonferroni test was conducted.

Effect sizes were calculated as partial eta-squared (η_p_^2^) and interpreted according to commonly accepted thresholds: 0.01 = small, 0.06 = medium, and ≥0.14 = large.

## 3. Results

### 3.1. Cognitive Function

#### 3.1.1. Vigilance Test

**Figure 2:**
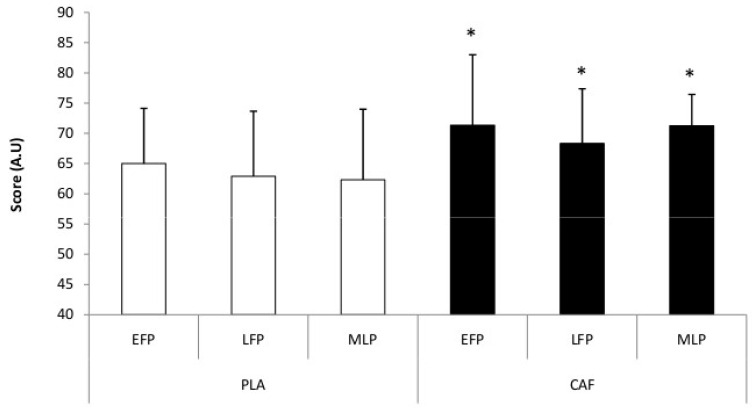
Variation in vigilance test scores during different phases of the menstrual cycle for placebo (PLA) and caffeine (CAF) conditions. EFP = early-follicular phase; LFP = late-follicular phase; MLP = mid-luteal phase. *: *p* < 0.05 to PLA condition.

Regarding VT data, our study only showed a significant condition effect (F_[1,11]_ = 14.11, *p* = 0.003, η_p_^2^ = 0.56, large effect). VT values were significantly higher in the CAF condition compared to the PLA across the different MCPs (*p* < 0.01). No significant difference was observed between MCPs in both conditions (*p* = 0.26) (Figure 2).

#### 3.1.2. Reaction Time

**Figure 3:**
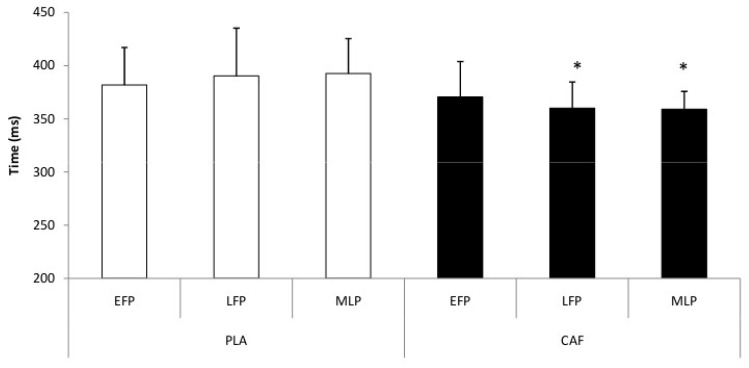
Change in reaction time during different phases of the menstrual cycle for both placebo (PLA) and caffeine (CAF) conditions. EFP = early-follicular phase; LFP = late-follicular phase; MLP = mid-luteal phase. *: *p* < 0.05 to PLA condition.

Statistical analysis of RTT data revealed a significant main effect of condition (F_[1,11]_ = 5.24, *p* = 0.04, η_p_^2^ = 0.32, large effect), indicating shorter RTT values following CAF ingestion compared with PLA. No significant condition × MCP interaction was detected (F_[2,22]_ = 0.98, *p* = 0.39, η_p_^2^ = 0.08, medium effect), suggesting that the effect of CAF did not differ across MCPs (Figure 3).

### 3.2. Physical Performance

#### 3.2.1. Counter-Movement Jump

**Figure 4:**
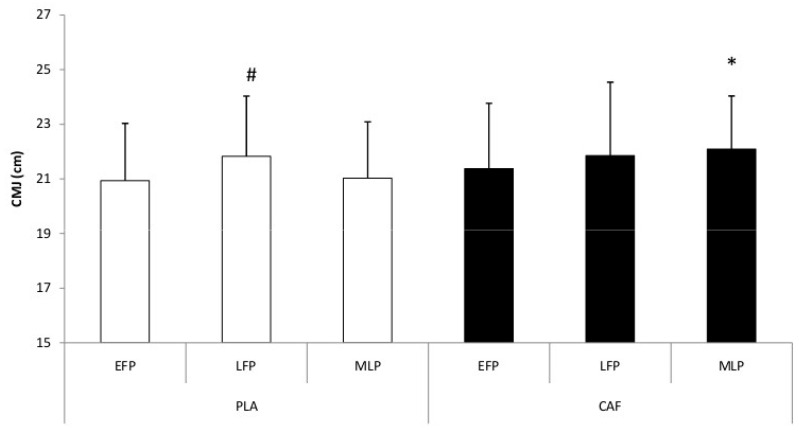
Change in counter-movement jump (CMJ) data during the different phases of the menstrual cycle for both placebo (PLA) and caffeine (CAF) sessions. EFP = early follicular phase; LFP = late follicular phase; MLP = mid-luteal phase. ^#^: *p* < 0.05 to EFP and MLP; *: *p* < 0.05 to PLA condition.

Statistical analysis revealed significant main effects of MCP (F_[2,22]_ = 4.19, *p* = 0.03, η_p_^2^ = 0.28, large effect) and condition (F_[1,11]_ = 3.85, *p* = 0.08, borderline significant, η_p_^2^ = 0.26, large effect), while the condition × MCP interaction was not significant (F_[2,22]_ = 2.22, *p* = 0.13, η_p_^2^ = 0.17, large effect). CMJ values were significantly higher in the LFP compared with both the MLP and EFP (*p* < 0.01) in the PLA condition. In addition, statistical analyses indicated higher CMJ performance in the MLP following CAF ingestion compared with PLA (Figure 4).

#### 3.2.2. Time to Exhaustion Test (TTE)

**Figure 5:**
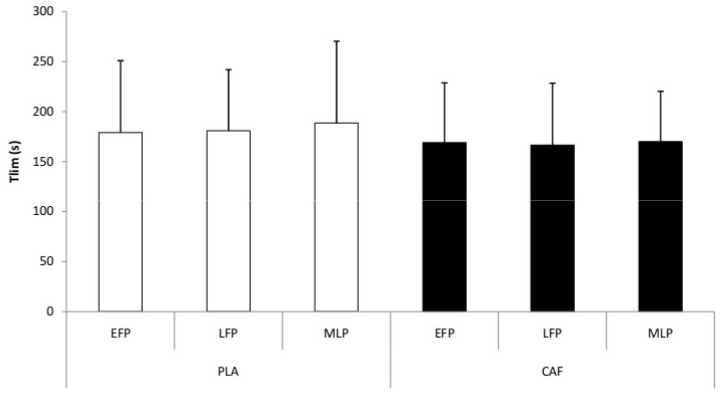
Change in the time to exhaustion during the different phases of the menstrual cycle for both the placebo (PLA) and caffeine (CAF) sessions across the different menstrual phases. EFP = early follicular phase; LFP = late follicular phase; MLP = mid-luteal phase.

Statistical analysis of TTE test data showed no significant interaction (condition × MCP) (F_[2,22]_ = 0.09, *p* = 0.91, η_p_^2^ = 0.008, negligible/trivial effect), condition (F_[1,11]_ = 0.44, *p* = 0.52, η_p_^2^ = 0.04, small effect), or MCP (F_[2,22]_ = 0.49, *p* = 0.62, η_p_^2^ = 0.04, small effect) effects. TTE data were similar between the CAF and PLA conditions and across the different MCPs (Figure 5).

#### 3.2.3. Repeated Sprint Test

Regarding RST data, statistical analysis revealed significant effects for sprint time (F_[9,99]_ = 74.5, *p* < 0.01, η_p_^2^ = 0.87, large effect) and MCP (F_[2,22]_ = 7.04, *p* < 0.01, η_p_^2^ = 0.39, large effect), with no significant interaction effect detected (F_[2,22]_ = 0.02, *p* = 0.98, η_p_^2^ = 0.002, negligible/trivial effect). Compared with the first sprint, peak power values decreased significantly from the 2nd to the 10th sprint in both conditions and across all MCPs (*p* < 0.05).

**Figure 6:**
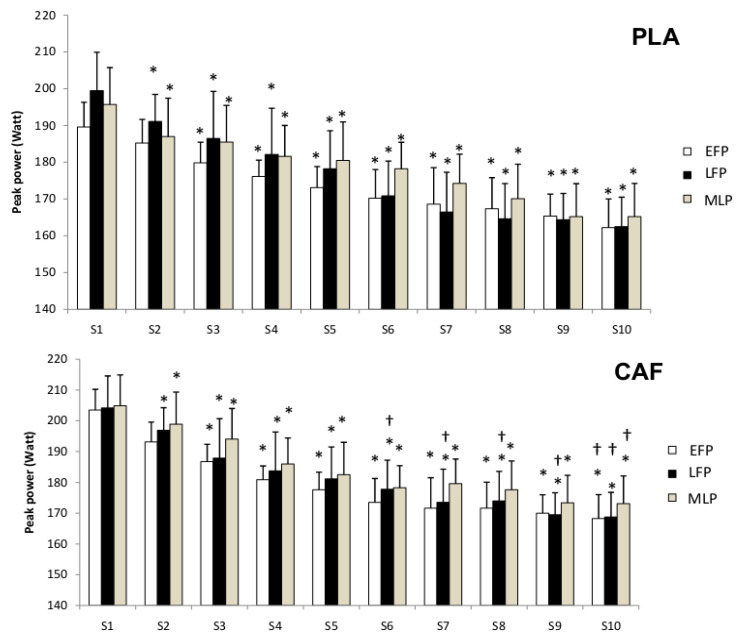
Peak power values during the repeated sprint test (RST) for both placebo (PLA) and caffeine (CAF) sessions across the different menstrual phases. EFP = early follicular phase; LFP = late follicular phase; MLP = mid-luteal phase. *: *p* < 0.05 to the 1st sprint; ^†^: *p* < 0.05 to PLA condition.

Compared to the PLA condition, peak power values were significantly higher in the LFP in the CAF condition from the 6th to the 10th sprint. Moreover, in the 10th sprint, peak power values were significantly higher across all MCPs in the CAF condition than in the PLA condition (Figure 6).

## 4. Discussion

The present study aimed to explore the effects of CAF supplementation on physical and cognitive performance across different phases of the MC in eumenorrheic female athletes. The present findings indicate that CAF supplementation improved cognitive performance, particularly vigilance (VT) and reaction time (RTT), compared with PLA, regardless of MCPs. In contrast, the effects of CAF on physical performance were more variable across the measured outcomes.

### 4.1. Effect on Cognitive Performance

The present study demonstrated that CAF supplementation enhanced RTT and VT scores across MC, without a significant difference across phases. This finding aligns with the established literature highlighting CAF’s ability to antagonize adenosine receptors (A1 and A2A), thereby increasing dopamine activity and reducing central fatigue. Increased dopamine availability [32] is known to improve attention, alertness, and executive function, which supports the enhanced cognitive outcomes observed in this study [33,34].

The absence of MC effect on cognitive outcomes suggests that the CAF’s central stimulatory effect may override the potential modulatory role of sex hormones on cognition. These results are consistent with prior findings that demonstrated CAF’s efficacy in improving reaction time and vigilance in both younger and older adults. Indeed, Swift et al. showed that 200 mg of caffeine enhanced RTT in healthy young people [35]. Moreover, Duncan et al. demonstrated that 3 mg/kg of CAF enhances concurrent anticipation timing performance in older women [36]. However, other studies have reported no effects of high CAF consumption (≥400 mg) on cognitive function [37,38]. Variability in CAF effects reported in other studies may be due to differences in dosage, mode of administration, individual sensitivity, and habitual CAF consumption [39,40]. While CAF consistently improved vigilance and reaction time across MCPs, it is important to note that the cognitive tasks employed were relatively simple. Therefore, these tasks may be less sensitive to subtle fluctuations in hormonal levels. Future studies using more complex or sensitive cognitive assessments may better elucidate the nuanced interactions between CAF, cognitive performance, and MCP.

### 4.2. Effect on Physical Performance

In the PLA condition, CMJ performance differed across MCPs, with higher values observed during the LFP, suggesting that jump performance may vary across the MC. This pattern is in agreement with previous work reporting superior CMJ performance during the LFP, possibly due to a more favorable hormonal environment for neuromuscular function during this phase.

In addition, CMJ values were higher in the CAF condition than in PLA during the MLP. However, because the interaction was not significant, this latter observation should be interpreted cautiously and not as definitive evidence of a phase-specific ergogenic effect of CAF. Rather, the present data suggest an overall beneficial effect of CAF on explosive performance, with MC-specific differences in baseline CMJ performance also contributing to the observed pattern.

Nevertheless, TTE performance was not significantly affected by either MCP or CAF supplementation. This aligns with studies showing variable and often minimal effects of CAF on aerobic endurance in female athletes, possibly due to inter-individual differences in CAF metabolism, training status, or baseline aerobic capacity. Additionally, TTE tests at 90% of maximal aerobic power may already reflect a ceiling effect in trained individuals, limiting the ergogenic potential of CAF.

On the other hand, sprint performance declined progressively across the 10 sprints under all conditions, consistent with the expected development of neuromuscular fatigue during repeated high-intensity efforts.

In the CAF condition, sprint performance was significantly enhanced, particularly during later sprints (6th–10th), and most notably during the LFP, when fatigue-related declines are typically more pronounced.

From a physiological view, MC exerts hormonal fluctuations that influence neuromuscular control, substrate metabolism, thermoregulation, and fatigue perception. Estradiol promotes increased glucose availability and central nervous system excitability, whereas progesterone exhibits sedative, thermogenic, and catabolic properties. Ergogenic effects of CAF likely stem from its ability to counteract inhibitory hormonal effects [41], stabilize neuromuscular transmission, and enhance both central and peripheral performance mechanisms. However, whether CAF can mitigate MCP-related variations requires confirmation in larger studies. It can be hypothesized that CAF’s ergogenic effects during the LFP may involve interactions with thermoregulation and substrate metabolism. Elevated estrogen can optimize body temperature and energy use, while CAF may help maintain neuromuscular performance by modulating metabolic and central nervous system activity. These mechanisms are speculative, warranting further research.

In the present study, CAF consistently enhanced vigilance across MC, representing the most robust finding. Improvements in reaction time and neuromuscular performance were more variable and phase-dependent, with no significant effect observed during EFP.

## 5. Practical Applications

The findings of this study highlight several practical implications for female athletes and their support teams. First, timing CAF intake, particularly during the MLP and LFP of the MC, may help counteract hormone-related declines in physical performance. This could be particularly relevant for sports that require high levels of explosive power and alertness. Coaches, trainers, and sport scientists should also consider incorporating individual MC tracking into performance planning. By aligning ergogenic strategies, such as CAF supplementation, with specific cycle phases, it may be possible to personalize interventions and optimize outcomes. The use of 400 mg of CAF was found to improve cognitive function and explosive physical performance, indicating that CAF can be a valuable tool in sports where rapid decision-making and neuromuscular power are critical.

Although the present findings suggest that CAF may enhance vigilance and reaction time in female athletes, these results are preliminary and should be interpreted with caution. Individual differences in CAF metabolism and MC responses may influence outcomes. Therefore, any recommendations for coaches or practitioners are provisional and context-dependent, and should primarily be used to guide future research rather than as definitive applied guidance.

## 6. Limitations and Future Directions

Although CAF showed some ergogenic effects on cognitive and physical performance, these findings are based on a final sample of 12 participants, and no formal a priori power calculation was conducted for the interaction models. Given the small sample size, the results should be interpreted cautiously, as they may reflect insufficient statistical power rather than the absence of true phase-dependent effects. The randomized crossover design allowed each participant to serve as their own control, which reduces inter-individual variability and partially improves the sensitivity of the analysis, but the findings remain exploratory. Future studies with larger samples are needed to confirm these results.

MCPs were confirmed using ovulation testing and serum hormone measurements; however, each phase is not hormonally homogeneous. Intra-phase variability in estrogen and progesterone levels may influence neuromuscular and cognitive performance and contribute to inter-individual differences in response to CAF. No analytical exploration was conducted to examine how individual hormone concentrations relate to performance outcomes. Future studies should consider more frequent hormonal sampling and hormone-performance correlation analyses to better account for this variability. This study only examined the short-term effects of CAF supplementation. Potential tolerance development, withdrawal symptoms, or long-term health impacts were not assessed. External factors such as psychological state, sleep quality, and nutritional intake were not strictly controlled, which could have influenced performance outcomes. Future research should address these factors, explore dose–response relationships, alternative delivery methods (e.g., caffeinated gum or beverages), and chronic supplementation effects to provide more comprehensive guidance. The use of a fixed CAF dose (400 mg) rather than a body-mass-adjusted dose may have contributed to variability in both physiological and cognitive responses. Future studies should consider individualized dosing to reduce inter-individual variability. Performance tests, including TTE at 90% maximal aerobic power, may have limited sensitivity to detect differences across CAF and PLA conditions or MCPs. Lower relative intensities or alternative endurance protocols could better assess CAF’s ergogenic potential. Finally, given the exploratory design, small sample size, and absence of ecologically valid performance measures, any applied recommendations regarding CAF use to mitigate MCP-related performance fluctuations should be considered preliminary. Larger studies with real-world settings are needed before definitive practical guidelines can be provided.

## 7. Conclusions

CAF supplementation has demonstrated the potential to enhance both cognitive and physical performance in female athletes, with improvements in VT observed consistently across the MC. Effects on RTT were phase-dependent, with no significant improvement during the EFP. While promising trends were observed for CMJ and RST, effects on endurance and repeated sprint outcomes remain less definitive. Individual variability in CAF sensitivity and phase-specific responses highlights the need for further research to optimize dosage and timing. As previously mentioned, given the small sample size and exploratory nature of the study, these findings should be interpreted cautiously and are intended to generate hypotheses for future research rather than provide definitive practical recommendations.

## Figures and Tables

**Table 1:** Descriptive data of the participants (mean ± standard deviation).

	EFP	LFP	MLP
Body mass (kg)	57.4 ± 8.9	58.0 ± 8.2	57.8 ± 8.9
TBW (%)	55.3 ± 3.6	54.72 ± 3.6	54.1 ± 3.7
HI			
Sleep	5 ± 1.1	4.5 ± 1.2	4.6 ± 1.3
Stress	2.8 ± 1.4	2.6 ± 1.1	2.5 ± 0.9
Fatigue	3.1 ± 1.7	3 ± 0.8	2.5 ± 1.0
DOMS	1.2 ± 0.9	1.1 ± 0.7	1.2 ± 0.8
Biochemical parameters			
Estrogen (pg/mL)	116.5 ± 23.1	226.1 ± 26.8	180.5 ± 11.5
Progesterone (ng/mL)	0.5 ± 0.3	0.7 ± 0.2	9.8 ± 2.1
Caffeine dose (mg/kg body mass)	7.1 ± 0.6	7.0 ± 0.5	6.9 ± 0.9

TBW: Total Body Water; EFP: Early follicular phase; LFP: Late follicular phase; MLP: Mid-luteal phase. HI (Hooper index): subjective self-reported scale ranging from 1 (very low) to 7 (very high) for sleep quality, stress, fatigue, and DOMS (Delayed Onset Muscle Soreness).

## Data Availability

The data presented in this study are available on request from the corresponding author due to privacy issues, subject to approval by the Regional Research Committee for Medical and Health Research Ethics and the local Data Protection Officer.

## Supplementary Materials

The following supporting information can be downloaded at: https://www.mdpi.com/article/10.3390/nu18101512/s1, Table S1: CONSORT 2025 checklist item description [42].
